# Stunting and its determinant factors among children aged 6–59 months in Ethiopia

**DOI:** 10.1186/s13052-017-0433-1

**Published:** 2017-12-19

**Authors:** Amare Tariku, Gashaw Andargie Biks, Terefe Derso, Molla Mesele Wassie, Solomon Mekonnen Abebe

**Affiliations:** 1 Department of Human Nutrition, Institute of Public Health, College of Medicine and Health Sciences, University of Gondar, Gondar, Ethiopia; 2 Department of Health Service Management and Health Economics, Institute of Public Health, College of Medicine and Health Sciences, University of Gondar, Gondar, Ethiopia

**Keywords:** Stunting, Children, Health and demographic surveillance system, Ethiopia

## Abstract

**Background:**

Though Ethiopia has implemented different nutritional interventions, childhood stunting on which literature is limited continues as a severe public health problem. Thus, this study aimed to investigate stunting and its determinants among children aged 6–59 months in the predominantly rural northwest Ethiopia.

**Methods:**

A community based cross-sectional study was conducted from May to June 2015 at Dabat Health and Demographic Surveillance System (HDSS) site. A total of 1295 mother-child pairs were included for analysis. An ordinal multivariable logistic regression analysis was carried out to identify the determinants of severe stunting. To show the strength of associations, both Crude Odds Ratio (COR) and Adjusted Odds Ratios (AOR) with a 95% Confidence Interval (CI) were estimated. Also, a *P*-value of <0.05 was used to declare statistical significance in the final model.

**Results:**

The overall prevalence of stunting among children aged 6–59 months was 64.5%, of which about 37.7% and 26.8% were moderately and severely stunted, respectively. Farming occupation of mother [AOR = 1.45; 95% CI: 1.08, 1.93], lack of postnatal vitamin-A supplementation [AOR = 1.54; 95%: 1.19, 2.00], poorer household wealth status [AOR = 2.07; CI: 1.56, 2.75] and accessing family food from farms [AOR = 1.44; 95% CI: 1.09, 1.89] were identified as the key determinants of severe stunting.

**Conclusion:**

In the district, the magnitude of stunting was a critical public health concern. Therefore, emphasis should be given to improving mothers’ postnatal vitamin A supplementation coverage and building knowledge about appropriate child feeding practices among farmer mothers and poorer households.

## Background

Stunting, low Height-for-Age Z-score (HAZ) is a global public health problem, affecting linear-growth potential of children. Worldwide, it affects 165 million (26%) children under 5 years [1]. The problem is graver in developing countries where it is the major contributor to child mortality [1, 2]. About 90% of the global stunted children live in Africa and Asia [2]; more than 40% are found in Sub-Saharan Africa, including Ethiopia [3, 4].

Childhood stunting (linear growth failure) is related to various adverse health consequences and irreversible damages. Stunting is correlated with poor developmental attainment [5] and intelligence in children [6, 7]. It is also documented that stunted children are less likely to be enrolled in school [8]. The risk of mortality and susceptibility to infections are also high among stunted children [1]. The consequences of child stunting also extend to adulthood. As an illustration, diminished productivity [7, 8], increased risk of excess weight gain and chronic non-communicable diseases in later life were frequently reported by earlier investigations [9, 10]. Moreover, stunting is a matter of great concern in terms of increased obstetric risks [7].

As the cause of stunting is complex and intertwined it needs further investigations because optimal child growth requires adequate nutrient supply and health appropriate care [11]. Earlier studies showed that feeding habits, health and socio-economic characteristics were significant determinants of child stunting. Inappropriate feeding practice, such as pre-lacteal feeding [12], non-exclusive breastfeeding, bottle feeding [13], low meal frequency and dietary diversity [14–16] as well as early or late initiation of complementary feeding [17] are significantly associated with stunting. Male sex [14] and frequent diarrheal episodes [12, 18] also increase the likelihood of stunting. Linear growth failure is documented in children whose mothers are illiterate, [19], old [19], work out of home [13, 19] and take no prenatal iron supplementation [15]. In addition, large family size and multiple siblings [13, 20, 21], food insecurity, poor wealth status, inadequate health care utilization and sanitary practices [17, 20, 22–26], unavailability of latrines [21, 27] and use of unprotected sources of drinking water [28] are the socio-economic determinates of stunting.

Despite a marked decline in the burden of undernutrition, stunting persisted as a severe public health problem in developing countries [1, 4, 29]. Also, nearly half (45%) of child mortality is associated with undernutrition [1]. In Ethiopia, a considerable numbers of such nutritional arrangements as the national nutritional program and the Infant and Young Child feeding Strategy were implemented in the last decades in order to protect children from the maladies of undernutrition [30, 31]. However, the burden of stunting remains a public health concern [4, 29]. Similarly, inappropriate maternal and child feeding practices are common in the country [4, 29]. Most pregnant and lactating mothers are suffering from different micronutrient deficiencies which affect the growth of fetuses and infants, respectively. Thus, the postnatal period is a window of opportunity to improve mothers’ micronutrient status, including the breast milk retinol level, through supplementation and other dietary approaches [32]. Thus, investigating the determinants of childhood stunting is of a paramount importance to design strategies to address the problem. However, literature is limited and even the available reports do not show the effect of independent variables on the level (severity) of stunting. Therefore, this study aimed to investigate stunting and associated factors among children aged 6–59 months using the ordinal logistic regression model.

## Methods

### Study setting

A community-based cross-sectional study was conducted from May to June 2015 at Dabat Health and Demographic Surveillance System (HDSS) site located in Dabat District, northwest Ethiopia. The livelihood of the inhabitants mainly depends on subsistence farming. The HDSS covers 67,385 people living in thirteen kebeles *(smallest administration unit in Ethiopia),* nine of which are rural.

### Sampling procedure

This study is part of a bigger survey entitled ‘Child Nutritional Status and Feeding Practice’. In the survey, eight kebeles were randomly selected from the total thirteen of the HDSS site. All children (6–59 months) living in these kebeles were included in the survey. Sample size was determined using Epi-info version 3.7 by considering the following assumptions; the prevalence of stunting in Amhara Region as 52% [4], 95% level of confidence, 5% margin of error, 10% non-response rate, and a design effect of 2. Thus, a minimum sample size of 844 was obtained. To improve the power of the study, 1295 children fulfilling the inclusion criteria were included in the study.

### Data collection and analysis

A structured interviewer-administered questionnaire was used to collect data. The English version questionnaire was translated to the national and local language. A pretest was done out of the study area before the actual data collection. A total of fourteen data collectors and three field supervisors were involved in the data collection. Training was given to data collectors and supervisors for 2 days.

The anthropometric assessment was done according to the standardized procedures stipulated by the Food and Nutrition Technical Assistance (FANTA) ‘Anthropometric Indicators Measurement Guide’ [33]. Height was measured using the seca vertical height scale (German, Serial No. 0123) standing upright in the middle of the board. The child’s head, shoulders, buttocks, knees, and heels touched the vertical board. The length of a child (aged 6–23 months) was measured using a horizontal wooden length board in recumbent position and read to the nearest 0.1 cm.

Anthropometric related data were transferred to the WHO Anthro-Plus software version 1.0.4 using Stat/Transfer version 9. The Z-scores of indices, Height-for-Age Z-scores (HAZ), were calculated using the WHO Multicenter Growth Reference Standard**.** The child was classified as severely stunted if his/her Z-score was less than −3 Standard Deviation (SD), moderately stunted (−3.00 ≤ HAZ < 2); otherwise he/she was defined as well-nourished if Z-score ≥ −2 SD [34].

Dietary diversity score (DDS) of a child was assessed by interviewing the mother to list all food and drink taken by the child in the 24 h preceding the survey. In case of mixed dish, the data collectors assisted mothers to list the ingredients of the food items, and using the standardized DDS tool, food items were categorized into seven food groups [35]. The DDS of four is considered as the minimum acceptable dietary diversity; accordingly a child with a DDS of less than four was classified as having poor dietary diversity; otherwise, it was considered to have good dietary diversity.

Data were entered into Epi-info version 3.5.3 and analyzed by using Stata version 12. Tables and graphs were used to present data, while frequencies and proportions were used to summarize the variables. The household wealth index was computed using a composite indicator for urban and rural residents by considering properties like, selected household assets and size of agricultural land. Using Principal Component Analysis (PCA), the factor score was summed and ranked into poor, medium, and rich. The ordinal logistic regression model was used to identify the determinants of severe stunting. A bivariable analysis was carried out to see the crude effect of each independent variable on severe stunting, and after that variables with *P*-values of <0.2 in the bivariable analysis were entered into the multivariable analysis. To show the strength of association, Adjusted Odds Ratio (AOR) with a 95% Confidence Interval (CI) was estimated. Also, a *P*-value of <0.05 was used to declare statistical significance in the final model. The parallel line assumption and the goodness-of-fit-test was checked, accordingly the model well fits the data.

## Results

A total of 1295 mother-child pairs were included in the study. The mean age (±SD) of children was 27.9 (±14.0) months, and 50.7% of whom were male. Nearly two-third, (56.2%) of the mothers were housewives, whereas about 27.1% were farmers. The majority of the households had male househeads (96.5%), and accessed food from farms (68.4%) (Table 1).

**Table 1:** Socio-demographic and economic characteristics of children (6–59 months) and their parents in the predominantly rural population of northwest Ethiopia, 2015

Characteristics	Frequency	Percent
Child age (in months)		
6–11	196	15.1
12–35	706	54.6
36–47	253	19.5
48–59	140	10.8
Mean age (±SD)	27.9 (±14.0)	
Sex of child		
Female	639	49.3
Male	656	50.7
Head of the household		
Female	45	3.5
Male	1250	96.5
Mothers age		
15–34 years	737	56.9
35–50 years	558	43.1
Marital status		
Currently unmarried	149	11.5
Currently married	1146	88.5
Religion		
Orthodox Christianity	1220	94.2
Others^a^	75	5.8
Household size		
≤ 4	470	36.3
5–7	632	48.8
8–10	193	14.8
Number of children under five		
1	101	7.8
2–4	1194	92.2
Maternal education		
No formal education	884	68.3
Primary education	189	14.6
Secondary education	222	17.1
Maternal employment status		
Housewife	728	56.2
Farmer	351	27.1
Others^b^	216	16.7
Paternal education		
No formal education	864	66.7
Formal education	431	33.3
Main source of family food		
Own production	886	68.4
Purchasing	364	28.1
Others^c^	45	3.5
Wealth status		
Poor	489	37.8
Medium	387	29.9
Rich	419	32.4
Health care access		
Good	1148	88.6
Poor	147	11.4

^a^Muslim and protestant Christianity

^b^Private business, students, servant, unemployed

^c^Food donating from government and families

Nearly half (46.1%) of the mothers took prenatal iron supplementation, but only few (1.7%) consumed extra meals during pregnancy. Furthermore, one-quarter (23.6%) of the mothers received postnatal vitamin-A supplementation. In this community, only half (51.5% and 51.2%) of the mothers gave colostrum and initiated breastfeeding within an hour of delivery, respectively. Moreover, about 62.4% of children were exclusively breastfed for the optimal duration of 6 months. Regarding complementary feeding practices, only 5.9% of children consumed a complementary food made of at least four food groups. The dietary pattern of the setting consisted of 91.9% and 73.3% of starchy staples and legumes, respectively, with 1.2%, 5.9%, and 1.3% of vitamin-A rich fruits and vegetables eggs, and other fruits and vegetables, in that order, in the 24 h preceding the date of the survey(Table 2).

**Table 2:** Maternal and child feeding practice in the predominantly rural population of northwest Ethiopia, 2015

Characteristics	Frequency	Percent
Extra food during pregnancy		
Yes	22	1.7
No	1273	98.3
Prenatal iron supplementation		
Yes	597	46.1
No	698	53.9
Colostrums		
Given to the child	667	51.5
Discarded	626	48.5
Breastfeeding initiation within 1 h		
Yes	663	51.2
No	632	48.8
Ever breastfed		
Yes	1287	99.4
No	8	0.6
Exclusive breastfeeding		
Yes	808	62.4
No	487	37.6
Pre-lacteal feeding		
Yes	369	28.5
No	926	71.5
Complementary feeding initiation		
Timely	740	57.1
Early	155	12
Late	400	30.9
Bottle feeding		
Yes	63	4.9
No	1232	95.1
Dietary diversity score		
< 4 food groups	1218	94.1
≥ 4 food groups	77	5.9
Starchy staples		
Yes	1190	91.9
No	105	8.1
Vitamin-A rich fruits and vegetables		
Yes	16	1.2
No	1279	98.9
Legumes, nuts and seeds		
Yes	949	73.3
No	346	26.7
Oils and fats		
Yes	834	64.4
No	461	35.6
Dairy products		
Yes	302	23.3
No	993	72.7
Meat, poultry, and fish		
Yes	164	12.7
No	1131	87.3
Egg		
Yes	77	5.9
No	1218	94.1
Other fruits and vegetables		
Yes	17	1.3
No	1278	98.7
Maternal vitamin A supplementation		
Yes	305	23.6
No	990	76.4
Deworming		
Yes	471	36.4
No	824	63.6
History of fever in the previous 2 weeks		
Yes	495	38.2
No	800	61.8
History of diarrheal attack in the previous 2 weeks		
Yes	242	18.7
No	1053	81.3

Most (61.3%) of the women used unprotected source of water for household consumption, and about one-fourth (26.3%) of mothers required more than 30 min to fetch water from the sources. Furthermore, most (92.4%) of the respondents didn’t treat water before consumption. Latrine was not available in 70.1% of the households (Table 3).

**Table 3:** Household related characteristics of the study participants in the predominantly rural population of northwest Ethiopia, 2015

Characteristics	Frequency	Percent
Source of drinking water		
Protected source	501	38.7
Unprotected source	794	61.3
Time to fetch water		
≤ 30 min	955	73.7
> 30 min	340	26.3
Water treatment		
Not at all	1196	92.4
Always	68	5.3
Sometimes	31	2.4
Availability of latrine		
Yes	387	29.9
No	908	70.1
Waste disposal		
Appropriate^a^	160	12.4
Inappropriate^b^	1135	87.6
Hand washing before feeding		
Not at all	12	0.9
Sometimes	41	3.2
Always	1242	95.9
Hand washing after toilet		
Not at all	140	10.8
Sometimes	222	17.1
Always	933	72

^a^Collected by municipality, buried and burned

^b^Dumped in street/open space, compound and river

The overall prevalence of stunting (<-2HAZ) among children aged 6–59 months was 64.5% [95% CI; 59.4, 69.6]. About 37.7% [95% CI; 32.5, 42.9%] and 26.8% [95% CI; 22.1, 31.5%] of children were moderately and severely stunted, respectively. Besides, severe stunting was observed among 22.2% and 20.3% children whose mothers didn’t receive postnatal vitamin-A supplementation and accessed family food mainly from their farms (own production), respectively (Table 4).

**Table 4:** Distribution of stunting by the selected characteristics among children aged 6–59 months in the predominantly rural population of northwest Ethiopia, 2015 (*N* = 1295)

Variables	Severity of stunting (Height-for-Age Z-score) (HAZ)			
	Severe stunting (HAZ < −3)	Moderate stunting (−3.00 ≤ HAZ < −2)	Normal (HAZ ≥ −2)	Total
Number of children under five				
1	21(1.6%)	34(2.6%)	46(3.6%)	101(7.8%)
2–4	326(25.2%)	454(35%)	414(32%)	1194(92.2%)
Wealth status				
Poor	187(14.4%)	189(14.6%)	113(8.7%)	489(37.7%)
Medium	90(7%)	148(11.4%)	149(11.5%)	387(29.9%)
Rich	70(5.4%)	151(11.7%)	198(15.3%)	419(32.4%)
Main source of family food				
Own production	263(20.3%)	332(25.6%)	291(22.5%)	886(68.4%)
Purchasing	66(5.1%)	142(10.9%)	156(12.1%)	364(28.1%)
Others	18(1.4%)	14(1.1%)	13(1%)	45(3.5%)
Maternal employment status				
Housewife	167(12.9%)	285(22%)	276(21.3%)	728(56.2%)
Farmer	146(11.3%)	122(9.4%)	83(6.4%)	351(27.1%)
Others	34(2.6%)	81(6.3%)	101(7.8%)	216(16.7%)
Health care access				
Good	295(22.7%)	431(33.3%)	422(32.6%)	1148(88.6%)
Poor	52(4%)	57(4.4%)	38(3%)	147(11.4%)
Source of drinking water				
Protected source	138(10.7%)	184(14.2%)	179(13.8%)	501(38.7%)
Unprotected source	209(16.1%)	304 (23.5%)	281(21.7%)	794(61.3%)
Maternal Vitamin A supplementation				
Yes	59(4.6%)	111(8.6%)	135(10.4%)	305(23.6%)
No	288(22.2%)	377(29.1%)	325(25.1%)	990(76.4%)
Exclusive breast feeding				
Yes	180(13.9%)	315(24.3%)	313(24.2%)	808(62.4%)
No	167(12.9%)	173(13.4%)	147(11.3%)	487(37.6%)
Complementary feeding initiation				
Timely	182(14.1%)	280(21.6%)	278(21.4%)	740(57.1%)
Early	39(3%)	59(4.6%)	57(4.4%)	155(12%)
Late	126(9.7%)	149(11.5%)	125(9.7%)	400(30.9%)
Dietary diversity score				
< 4 food groups	334(25.8%)	463(35.8%)	421(32.5%)	1218(94.1%)
≥ 4 food groups	13(1%)	25(1.9%)	39(3%)	77(5.9%)

After controlling for potential confounders, the result of the multivariable ordinal logistic regression analysis revealed that wealth status, maternal occupation, source of family food, and postnatal maternal vitamin-A supplementation were significantly associated with severe stunting. Accordingly, the odds of severe stunting were higher among children whose mothers were farmers [AOR = 1.45; 95% CI: 1.08, 1.93] and didn’t receive postnatal vitamin-A supplementation [AOR = 1.54; 95%: 1.19, 2.00]. Likewise, being members of poorer households [AOR = 2.07; CI: 1.56, 2.75] and medium wealth status households [AOR = 1.37; 95% CI: 1.03, 1.83] was more associated with increased odds of childhood severe stunting than being members of richer households. More severely stunted growth was observed among children from households which accessed family food mainly from farms (own production) than those who mainly accessed from the market, by purchasing [AOR = 1.44; 95% CI: 1.09, 1.89](Table 5).

**Table 5:** An ordinal logistic regression showing the determinants of severe stunting among children aged 6–59 months in the predominantly rural population of northwest Ethiopia, 2015

Variable	Frequency	Severe stunting n (%)	AOR [95% CI]
Number of under five children			
1	101	21 (20.8)	1
2–4	1194	326 (27.3)	0.96 (0.61, 1.52)
Household size			
≤ 4	470	111 (23.6)	1
5–7	632	183(29)	1.10 (0.85, 1.42)
8–10	193	53 (27.5)	0.89 (0.61, 1.30)
Wealth status			
Poor	489	187(38.2)	2.07 (1.56, 2.75)^a^
Medium	387	90 (23.3)	1.37 (1.03, 1.83)^a^
Rich	419	70 (16.7)	1
Main source of family food			
Own production	886	263 (29.7)	1.44 (1.09, 1.89)^a^
Purchasing	364	66 (18.1)	1
Others	45	18(40)	1.74 (0.94, 3.23)
Maternal education			
No formal education	884	256 (29.0)	1.24 (0.85, 1.79)
Primary education	189	54(28.6)	1.29 (0.86, 1.93)
Secondary education	222	37 (16.7)	1
Maternal employment status			
Housewife	728	167 (22.9)	1
Farmer	351	146 (41.6)	1.45 (1.08, 1.93)^a^
Others	216	34 (15.7)	1.02 (0.71, 1.49)
Paternal education			
No formal education	864	254 (29.4)	1.08 (0.84, 1.40)
Formal education	431	93 (21.6)	1
Health care access			
Good	1148	295 (25.6)	1
Poor	147	52(35.4)	1.38 (0.99, 1.91)
Source of drinking water			
Protected source	501	138 (27.5)	1
Unprotected source	794	209(26.3)	0.92 (0.72, 1.17)
Availability of latrine			
Yes	387	93(24)	1.15 (0.90, 1.48)
No	908	254(28)	1
Maternal vitamin A supplementation			
Yes	305	59 (19.3)	1
No	990	288 (29.1)	1.54 (1.19, 2.00)^a^
Breastfeeding initiation within 1 h			
Yes	663	161 (24.3)	1
No	632	186(29.4)	0.90 (0.71, 1.16)
Exclusive breastfeeding			
Yes	808	180(22.3)	1
No	487	167(34.3)	1.31(0.94, 1.83)
Complementary feeding initiation			
Timely	740	182(24.6)	1
Early	155	39 (25.2)	0.80 (0.54, 1.19)
Late	400	126 (31.5)	1.01 (0.72, 1.41)
Dietary diversity score			
< 4 food groups	1218	334(27.4)	1.44 (0.91, 2.28)
≥ 4 food groups	77	13 (16.9)	1

^a^significant at a *P*-value of < 0.05

## Discussion

In the present study, the magnitude of overall and severe stunting among children was higher than the recent Demographic and Health Survey Reports of Ethiopia (overall stunting 44%, severe stunting 21%)) [4] and Nepal (overall stunting 40.6%, severe stunting 15.9%) [25]. The discrepancy could be explained by the depth of the studies in that the latter reports were national with larger number of children. In contrast, this study was done only in the rural areas of northwest Ethiopia. In fact, because of poor dietary habit, nutritional awareness [4], and limited allocation of health care resources [4, 36], stunting is more common in rural areas [37]. On the other hand, studies in developing countries claimed that stunting is less common in infants aged less than 6 months as they are on breastfeeding [38]. However due to inappropriate complementary feeding practices and increased nutritional demand, the risk of impaired linear growth becomes higher after the sixth month [39]. Therefore, the high prevalence of overall and severe stunting in this study could also be related to the exclusion of infants aged less than 6 months, while they were included in the other studies.

Similarly, our finding was higher than that of another local study in Bule Hora district, south Ethiopia (overall stunting 47.6%, severe stunting 20.2%) [12]. The difference could be related to variations in the livelihood of the inhabitants; livestock and cash crops are the major economic sources in Bule Hora, whereas it is subsistence farming in the current study area. Given that, children of the former study area might have protein-rich animal food which is protective against the risk of stunting [40].

The result of the ordinal multivariable analysis showed that the odds of being severely stunted were higher among children from poorer and medium wealth status families. This is due to the fact that compared to the better-offs poorer households are incapable of purchasing nutritionally adequate and diversified food for their children. In fact, insufficient food intake, exposure to infections, and lack of access to basic health services are associated with stunning [27]. Moreover, the finding was supported by those of previous studies in developing countries [14, 25, 27].

Also, children of farmer mothers were at higher risk of facing severely stunted growth than children of housewife mothers. Parallel findings were also reported from Southern Ethiopia [13, 19]. Obviously, outdoor worker mothers do not have enough time to care and appropriately feed their children compared to housewife mothers. For that reason, sub-optimal breastfeeding and complementary feeding, the major risk factors of stunting are higher among outdoor worker mothers [41, 42]. However, a study in South Africa showed that children of outdoor worker mothers were less likely to be stunted [43]. That result suggested the positive effect of maternal employment in enhancing child nutritional status, mainly through improving household income, food security status, and utilization of health services [44, 45].

Surprisingly, higher odds of severe stunting were noted among children from households which accessing their food from farm (own production) compared to those from the households accessed their food through purchasing and donation. Though having a farm has its own role in improving e household food access [46], in many parts of Ethiopia farmers do not consume the produced food items, particularly animal based food stuff. In addition, almost all farmers living in rural areas of the country are more vulnerable to undernutrition [45].

To sum up, children whose mothers received no postnatal vitamin-A supplementation more odds of severe stunting than their counterparts. Improving vitamin-A status of children is one of the proven child survival strategies; it is found to especially reduce the risk of morbidity and mortality from infectious diseases [47, 48]. Frequent episodes of infectious diseases, such as diarrhea and respiratory tract infections are strongly associated with a higher risk of stunting [49]. In Ethiopia, most pregnant mothers suffer from vitamin-A deficiency [32], and have poor dietary intake of vitamin-A [4]. As a result, the postnatal period is a window of opportunity to improve mother’s vitamin-A status thereby increasing the breast milk retinol level. This way, breastfed infants get an adequate amount of vitamin-A which further helps to reduce the risk of infectious disease episodes through boosting immunity.

## Conclusion

Stunting is a severe public health problem in the predominantly rural northwest Ethiopia. Mother’s occupation, postnatal vitamin-A supplementation, source of family food and household wealth status were identified as determinants of severe stunting. Therefore, emphasis should be given to improving maternal postnatal vitamin A supplementation coverage and building knowledge on appropriate feeding practices, particularly among farmer and poorer households.

## Abbreviations

AOR: Adjusted Odds Ratio
CI: Confidence Interval
COR: Crude Odds Ratio
DDS: Dietary Diversity Score
FANTA: Food and Nutrition Technical Assistance
HDSS: Health and Demographic Surveillance System
IYCF: Infant and Young Child Feeding
PCA: Principal Component Analysis
SD: Standard Deviation
WHO: World Health Organization
